# The effect of different intensity physical activity on cardiovascular metabolic health in obese children and adolescents: An isotemporal substitution model

**DOI:** 10.3389/fphys.2023.1041622

**Published:** 2023-02-15

**Authors:** Youxiang Cao, Lin Zhu, Zekai Chen, Li Zhanquan, Weijun Xie, Manna Liang

**Affiliations:** ^1^ Graduate Department of Guangzhou Sport University, Guangzhou, China; ^2^ School of Sport and Health, Guangzhou Sport University, Guangzhou, China; ^3^ Key Laboratory of Education Department of Guangdong Province, Guangzhou, China

**Keywords:** physical activity, cardiovascular metabolic health, obesity, isotemporal substitution model, children/adolescents

## Abstract

**Objective:** This study’s purpose was to investigate the effects of different intensities of physical activity on cardiovascular metabolism in obese children and adolescents based on an isochronous replacement model.

**Methods:** A total of 196 obese children and adolescents (mean age, 13.44 ± 1.71 years) who met the inclusion criteria and attended a summer camp from July 2019 to August 2021 were recruited for this study, and all subjects wore a GT3X + triaxial motion accelerometer uniformly around the waist to record physical activity levels. We collected the subjects’ height, weight, and cardiovascular risk factors such as waist circumference, hip circumference, fasting lipids, blood pressure, fasting insulin, and fasting glucose before and after 4 weeks of camp and constructed cardiometabolic risk score (CMR-z). We analyzed the effects of different intensities of physical activity on cardiovascular metabolism in obese children using isotemporal substitution model (ISM).

**Results:** After 4 weeks, cardiovascular risk factors such as body weight, waist circumference, triglyceride, and total cholesterol were reduced in adolescents with obesity (*p* <0.01), and CMR-z was also reduced (*p* <0.01). ISM analysis revealed that all sedentary behavior (SB) replacement with 10 min of light physical activity (LPA) reduced CMR-z [β = −0.10, 95% CI (−0.20, −0.01)]; 10-min of moderate physical activity (MPA) replacement of SB reduced CMR-z [β = −0.32, 95% CI (−0.63, −0.01)]; 10-min of vigorous physical activity (VPA) replacement of SB reduced CMR-z [β = −0.39, 95% CI (−0.66, −0.12)].

**Conclusion:** Replacement of SB with 10 min of LPA, MPA, and VPA were all effective in improving cardiovascular risk health, respectively, but MPA or VPA was more effective.

## 1 Introduction

As of 2015, there were approximately 107 million obese children and adolescents worldwide, with more than 70 countries experiencing a twofold increase in the prevalence of childhood and adolescent obesity compared with 1970 (Collaborators et al., 2017). Along with the increased prevalence of obese children and adolescents, cardiovascular disease has become a substantial social problem and has significantly increased the medical burden on society (Gortmaker et al., 1993; Lightwood et al., 2009). Studies have found that obese children and adolescents have a significantly higher risk of developing cardiovascular risk factors such as insulin resistance, type 2 diabetes mellitus, dyslipidemia, and hypertension than children and adolescents of normal weight (Tounian et al., 2001; Järvisalo et al., 2002; Kit et al., 2015; American Diabetes Association, 2017), and obesity in children and adolescents can lead to a significantly higher incidence of cardiovascular disease in adulthood (Tirosh et al., 2011; Berenson and Bogalusa Heart Study group, 2012; Drozdz et al., 2021). An important cause of childhood obesity is decreased physical activity and increased sedentary behavior (SB) time. Several extensive research studies have shown that SB time, especially screen time, increases every year in children and adolescents and that the attainment rate of physical activity is low, especially among obese children (Cooper et al., 2015; Larouche et al., 2017; Larouche et al., 2019; Zhu et al., 2019).

Total physical activity (TPA) includes SB, light physical activity (LPA), moderate physical activity (MPA), and vigorous physical activity (VPA); the duration of SB is strongly associated with cardiovascular risk (Buman et al., 2014; Lavie et al., 2019). Increasing moderate-intensity physical activity can offset the increased cardiovascular risk caused by sedentary hypermobility (Ekelund et al., 2016). In a prospective meta-analysis study, Skrede et al. (2019) found that moderate-vigorous physical activity (MVPA) levels in children and adolescents were significantly and negatively associated with the risk of cardiovascular morbidity. Skrede et al. (2017) found that increasing the duration of MPA was effective in reducing cardiometabolic risk in children. The World Health Organization (WHO) also recommends that children and adolescents accumulate at least 60 min of moderate-to-vigorous intensity physical activity per day (Chaput et al., 2020).

Despite the effectiveness of MVPA in reducing cardiovascular disease risk, previous studies have usually examined different intensities of physical activity and SB as independent factors, ignoring the interactive effect of SB with different PA on cardiovascular risk. The 24 h of a day mainly includes sleep, SB, LPA, MPA, and VPA, thus adding more of one activity requires a corresponding reduction of another one (Chastin et al., 2015). The isotemporal substitution model (ISM) is a novel statistical research method in epidemiological studies that can effectively explore the effects on health when PA varies in intensity and is also easy to apply in clinical practice (Mekary et al., 2009). Lemos et al. (2021) found that decreasing SB and increasing physical activity practice can effectively improve cardiorespiratory fitness in children. Although ISM can explore the relationship between a specific physical activity and health outcomes, the results were not always consistent. Sardinha et al. (2017) found only MVPA can effectively promote health, not for LPA. Sun et al. (2020) also found that reallocating SB into MVPA can effectively improve cardiorespiratory fitness. LPA is an important component of daily physical activity (Carson et al., 2013), whether reallocating SB into LPA is effective in improving health remains to be further investigated. At present, there are relatively few studies based on ISM on Cardiometabolic risk (CMR) in obese children. Therefore, a specific, ISM-based approach is also needed to explore the effects of different intensities of PA on CMR when substituted for one another, providing a highly valuable quantitative basis for public health policy making.

The purpose of this study was to investigate the effects of SB replacement with different intensities of PA on cardiovascular risk factors and cardiovascular risk in obese pediatric adolescents based on an isochronous replacement model.

## 2 Participants and Methods

### 2.1 Participants

**FIGURE 1:**
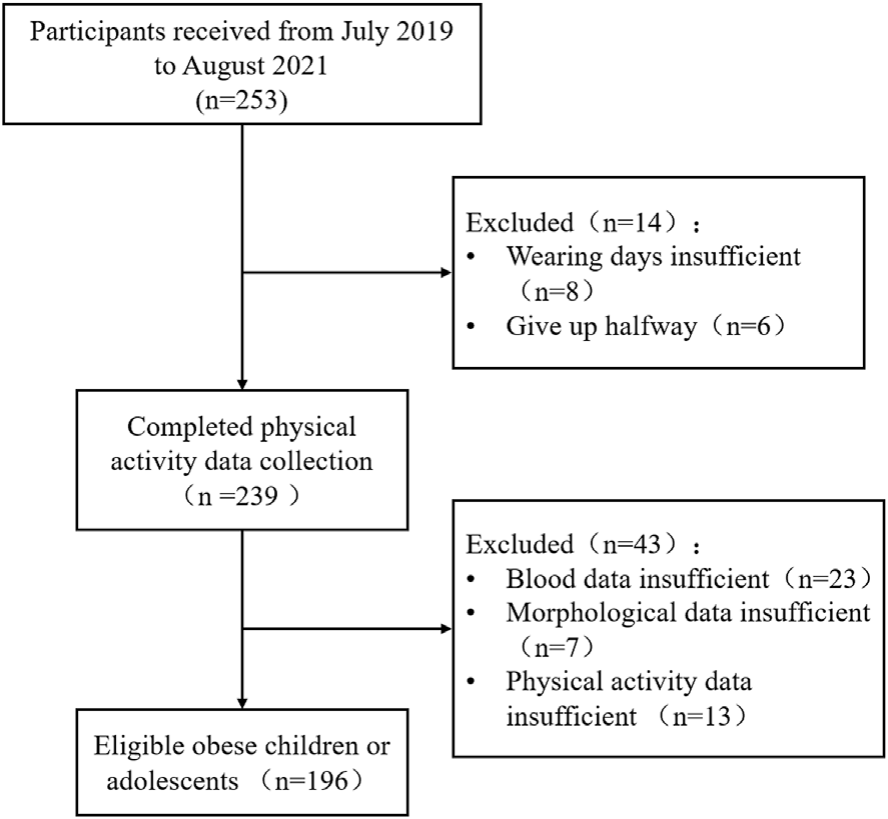
Participants screened flow chart.

The participants in this study were obese children and adolescents who participated in a summer camp between July 2019 and August 2021 (Figure 1; Table 1). The inclusion criteria for subjects were that they: 1) met the obesity identification criteria in Chinese children and adolescents (GoCOT and Force, 2004), 2) could cooperate with researchers to complete relevant tests, and 3) could participate in sports training normally. Exclusion criteria were: 1) pathological obesity (BMI ≥40 kg/m^2^), 2) being unable to participate in normal exercise training, 3) taking medication for obesity or being medically diagnosed as unfit for exercise, or 4) suffering from psychiatric disorders. All subjects and parents were informed of the possible risks associated with the experiment and signed an informed consent form before the experiment. The subjects finished the self-reported pubertal development scale, which can effectively evaluate the puberty development stage of Chinese children and adolescents (Zhu and Chen, 2012). This study was approved by the Ethics Committee of Guangzhou Sports Institute (Approval number: 2018LCLL-008).

**TABLE 1:** Baseline characteristics of Participants.

	Total (*n* = 196)	Boys (*n* = 106)	Girls (*n* = 90)	*P*
Age (years)	13.44 ± 1.71	13.23 ± 1.83	13.69 ± 1.53	0.06
Body Weight (kg)	81.97 ± 15.16	83.71 ± 16.27	79.91 ± 14.40	0.09
Height (cm)	163.56 ± 8.66	165.18 ± 9.48	161.65 ± 7.23	<0.01
BMI (kg/m^2^)	30.52 ± 4.22	30.54 ± 4.11	30.50 ± 4.36	0.94
REE (kcal/d)	1758.05 ± 245.51	1864.50 ± 232.36	1632.67 ± 197.41	<0.01

BMI, body mass index; REE, rest energy expenditure; *p*-values refer to systematic differences between boys and girls.

This study calculated the overall sample size based on Tabachnick and Fidell’s formula: n > 50 + 8 × m (m is the number of independent variables) (Tabachnick and Fidell, 2007). There are seven variables in this study (*m* = 7), thus according to the equation the effective sample size should be at least 106 (n >50 + 8 × 7).

### 2.2 Body morphology and cardiovascular risk factor measurements

The subjects’ basic information was collected, such as height, weight, age, gender, waist circumference (WC), and hip circumference (HC). Height measured with height meter that was accurate to 0.1 cm (RuKe RK001, Zhejiang, China); we measured weight using an electronic scale that was accurate to 0.1 kg. WC and HC were measured by using a non-elastic leather ruler (RHOS, GC059, Guangdong, China), which was accurate to 0.1 cm and placed directly on the skin at the midpoint between the lower border of the rib cage and the iliac crest of the waist (Physical status, 1995) and at the maximum extension of the buttocks for the hip (Wang et al., 2000). Blood pressure were measured by electronic sphygmomanometer (Omron, HEM-1020, Japan) in a sitting position with at least 5 min of quiet rest before measurement. Each subject was measured 3 times, with at least 1 min between each measurement, and took the average value. If the difference between any two measurements was greater than 10 mmHg, the closest two measurements were taken to calculate the average value as the final blood pressure value. To guarantee the quality control of the measurements, BW, height, HC, and WC were measured twice by a single evaluator and average value was taken: The values of the technical error of measurement range from 1.0 to 2.0%. Before the study begins, a specialist in anthropometric measurements provided a theoretical and practical training to the evaluator, and dozens of children and adolescents were measured at the same time by the specialist and the evaluator to confirm that the measurements were accurate.

Fasting venous blood samples were collected in the early morning (fasting for more than 12 h) and extracted the supernatant after low-temperature centrifugation and stored it in a refrigerator at −80°C for measurement. Fasting plasma glucose was measured by the glucose oxidase method (Beijing North Biotechnology Invest, China) with the sensitivity was 10 μmol/L. Fasting plasma insulin were determined by electrochemiluminescence method (Guangzhou Bioman Biotechnology Invest, China) with the sensitivity was 1.0 pmol/L. Total cholesterol (TC), triglyceride (TG), high-density lipoprotein cholesterol (HDL-c), low-density lipoprotein cholesterol (LDL-c) by fully automatic biochemical analyzer (Beckman Automatic Biochemical Analyzer, AU5800, United States).

### 2.3 Cardiovascular metabolic risk Z-Score

To assess cardiometabolic risk in children and adolescents, fasting insulin (FINs), FPG, WC, HDL-c, TC, and mean artery pressure (MAP) were standardized and added together to construct a cardiometabolic risk score (CMR-z, see calculation Eq. 1) (Andersen et al., 2006; Cliff et al., 2014; Väistö et al., 2014; Väistö et al., 2019). MAP is expressed as the mean of the sum of systolic and diastolic blood pressure; because elevated HDL-c is a protective factor for cardiometabolic risk, it is processed by multiplying by −1 and then normalizing. A larger CMR-z score indicates greater cardiovascular risk (Ekelund et al., 2007).

$$CMR-z=Z_{\left(FINs\right)}+Z_{\left(FPG\right)}+Z_{\left(WC\right)}+Z_{\left(HDL-c\right)}+Z_{\left(TC\right)}+Z_{\left(MAP\right)}$$ (1)

### 2.4 Exercise intervention program

The exercise intervention was 240 min per day: 120 min in the morning and 120 min in the afternoon, from 09:00–11:00 and from 15:00–17:00. Participants warmed up for 15 min before each exercise training and stretched and relaxed for 15 min after the training. The forms of exercise training were mainly different forms of aerobic exercises, such as running, jumping, gymnastics, badminton, rugby, basketball, etc., Participants wore a Polar heart rate band (Polar, OH1, Finland) during exercise training to monitor the intensity of exercise, and the overall exercise intensity was maintained at between 60% and 70% of maximal heart rate.

### 2.5 Diet program

A specialized dietitian developed the participants’ diet, prepared the meals, and recorded both the type and weight of the food. During the exercise intervention, daily energy supply according to the Molnár equation (Molnár et al., 1995) after calculating the resting energy expenditure (REE) of obese children. Dietary caloric supply based on the resting metabolic level. The dietitian matched the diet types according to the Dietary Guidelines for Chinese Residents, in which the ratio of energy supply was 30%:40%:30% for morning, midday, and evening meals; the ratio of caloric distribution of carbohydrates, protein, and fat in the diet was 55%–65%:20%–30%:10%–15%. Mainly, the food types included fresh vegetables, fruits, eggs, meat, and cereals.

### 2.6 Physical activity monitoring and data interception

ActiGraph GT3X + tri-axial motion accelerometer (ActiGraph, Pensacola, United States) were used to record the physical activity of the subjects during training. Basic information (height, weight, date of birth, etc.,) was entered into the ActiGraph GT3X + tri-axial exercise accelerometer using Actilife software (ActiLife 6, Pensacola, United States); The purpose, method, and precautions for wearing the device were explained before distributing it. The ActiGraph GT3X + tri-axial motion accelerometer was set to a sampling frequency of 30 Hz and a sampling interval of 60 s. Participants wore the device from 09:00 to 21:00 each day for a total of 12 h. Each participant wore the device at least 3 days per week for at least 2 weeks.

Actilife software intercepted the motion intensity based on the recorded vector magnitude values of ActiGraph GT3X+, and the formula for calculating vector magnitude is shown in Eq. 2, where Axis 1, Axis 2, and Axis 3 represent the frontal, sagittal, and vertical axes recorded by ActiGraph GT3X+, respectively. The cut point values for SB are 0–99 count/min; 100–3686 count/min for LPA, 3687–5246 count/min for MVPA, and ≥5247 count/min for VPA (the results of this study have not yet been published).

$$Vector\;Magnitude=\sqrt{{Axis1}^{2}+{Axis2}^{2}+Axis3^{2}}$$ (2)

### 2.7 Model building

In this study, three different models, single model, partition model, and ISM, were used to examine the effects of physical activity of different intensities on CMR-z after correcting for age and gender, with sedentary time, low physical activity time, moderate-intensity physical activity time, and high-intensity physical activity time as independent variables. We examined the effect of different intensities of physical activity on CMR-z using sedentary time, low physical activity time, moderate physical activity time, and high-intensity physical activity time as independent variables and CMR-z change as the dependent variable after correcting for age and gender. In this study, 10 min/d was used as a replacement unit of analysis because 10 min/d is not only the smallest unit of health benefit to an individual (WHO Guidelines Approved by the Guidelines Review Committee, 2010) but also the shortest duration of health risk from sedentary and less active behavior (King et al., 2016). In addition, this study further analyzed the effect of replacing 20 min, 30 min, 40 min, 50 min, and 60 min on CMR-z.

The single model evaluates the effect of a certain intensity of physical activity on outcome indicators alone, without considering other physical activity intensity times. For example, to evaluate the effect of LPA on CMR-z, ΔCMR-z = (β0) LPA + (β1) covariate.

The partition model is a statistical model in which all activity times are included in the analysis, and the coefficient of each physical activity represents the effect of changing the time of a certain physical activity on the outcome indicator, provided that the intensity of other physical activities remains constant. For example, ΔCMR-z = (β0) SΒ + (β1) LPA + (β2) MPA + (β3) VPA + (β4) covariates.

The ISM is to replace one physical activity intensity time with another physical activity intensity time in the same time period to calculate the effect of the replacement on the outcome index, which is a dummy effect estimation based on the premise of constant physical activity time. For example, ΔCMR-z = (β0) SΒ + (β1) LPA + (β2) MPA + (β3) total physical activity time + (β4) covariates whereas β0 in the formula represents the effect on the outcome indicator caused by replacing VPA with SB per unit of time.

### 2.8 Statistical analysis

The R (version 4.2.0, https://www.r-project.org/) and SPSS 25.0 (SPSS, Inc., Chicago, IL, United States) software were used for the statistical analysis of the data. The data were expressed as mean ± standard deviation (mean ± SD) and analyzed for before and after differences using one-way ANOVA. The Shapiro–Wilk test was used to verify normal data distribution. Δ + index represent the difference between pre- and post-intervention. We analyzed the correlation between CMR-z and time spent in physical activity of different intensities using Person correlation analysis; *p* <0.05 indicates statistical significance.

## 3 Results

### 3.1 Baseline characteristics of participants

A total of 196 eligible subjects, 106 boys and 90 girls, were included in the final analysis. The body weight of the boys was greater than that of the girls, but there was no other difference between them (*p* >0.05). Height and REE were higher in boys than in girls (*p* <0.01), as shown in Table 1.

### 3.2 Daily physical activity

The average daily physical activity of all subjects was 588 ± 67 min, of which 274 ± 53 min were SB, which was much higher than MPA and VPA. The SB duration was longer in girls than boys (*p* = 0.14) whereas the VPA duration was longer in boys but with no differences (*p* = 0.10), as shown in Table 2.

**TABLE 2:** Participants’ average daily physical activity of different intensities.

	Total (*n* = 196)	Boys (*n* = 106)	Girls (*n* = 90)	*P*
SB	274 ± 53	269 ± 52	280 ± 54	0.14
LPA	241 ± 46	242 ± 49	240 ± 42	0.81
MPA	38 ± 14	40 ± 15	35 ± 13	0.02
VPA	35 ± 16	36 ± 17	33 ± 15	0.10
TPA	587 ± 67	587 ± 67	588 ± 66	0.90

LPA, light physical activity; MPA, moderate physical activity; SB, sedentary behavior; VPA, vigorous physical activity; TPA, total physical activity; *p*-values refer to systematic differences between boys and girls.

### 3.3 Changes in morphological indicators and CMR factors

After the 4-week intervention, subjects showed significant changes in most indicators. Subjects’ morphological indicators, such as weight, BMI, HC, and WC, were improved (*p* <0.01). Cardiovascular risk factors FINs, TG, TC, systolic blood pressure, diastolic blood pressure, and MAP were reduced after the intervention (*p* <0.01), but FPG and HDL-c did not show significant changes (*p* >0.05); CMR-z showed a significant reduction (*p* = 0.02) (Table 3).

**TABLE 3:** Changes in morphological indicators and CMR factors.

	Pre-intervention	Post-intervention	Changes	*P*
Body weight (kg)	81.97 ± 15.52	74.48 ± 14.29	−7.49 ± 2.97	<0.01
BMI	30.52 ± 4.22	27.71 ± 3.87	−2.81 ± 1.15	<0.01
HC (cm)	107.25 ± 9.43	101.19 ± 9.37	−6.06 ± 2.99	<0.01
WC (cm)	99.50 ± 12.00	91.58 ± 10.96	−7.93 ± 4.40	<0.01
FINs (mIU/L)	13.67 ± 8.68	10.67 ± 6.26	−3.01 ± 7.58	<0.01
FPG (mmol/L)	4.74 ± 0.79	4.67 ± 0.82	−0.07 ± 0.92	0.38
TG (mmol/L)	1.24 ± 0.58	0.79 ± 0.36	−0.45 ± 0.50	<0.01
TC (mmol/L)	4.71 ± 1.02	3.74 ± 0.75	−0.97 ± 0.72	<0.01
HDL-c (mmol/L)	1.15 ± 0.24	1.16 ± 0.26	−0.01 ± 0.23	0.58
SBP (mmHg)	113.27 ± 10.53	105.65 ± 10.36	−7.62 ± 9.45	<0.01
DBP (mmHg)	68.75 ± 8.74	63.04 ± 8.42	−5.71 ± 8.67	<0.01
MAP (mmHg)	91.01 ± 8.26	84.34 ± 8.15	−6.67 ± 7.45	<0.01
CMR-z	1.39 ± 2.50	−1.39 ± 2.21	−2.78 ± 2.33	0.02

BMI, body mass index; CMR-z, Cardiometabolic risk Z-score; DBP, diastolic blood pressure; FINs, Fasting Insulin; FPG, fasting plsma glucose; HC, hip circumference; HDL-c, High-density Lipoprotein Cholesterol; MAP, mean artery pressure; SBP, systolic blood pressure; TG, triglyceride; TC, total cholestero; WC, waist circumferencel.

### 3.4 Correlation between CMR factor changes with different intensities of physical activity and TPA

After 4 weeks of intervention, Pearson correlation analysis of CMR factors changes, CMR-z with different physical activity intensities, and TPA revealed that FINs, FPG, TC, and TG were all significantly negatively correlated with LPA, MPA, and VPA and significantly positively correlated with SB. The CMR-z analysis also found significant negative correlations with LPA, MPA, and VPA and positive correlations with SB (Table 4).

**TABLE 4:** Correlation between CMR factors changes with different intensities of physical activity and TPA.

	ΔFINs	ΔFPG	ΔMAP	ΔTC	ΔWC	ΔHDL-c	ΔCMR-z
SB	0.16*	0.06	0.11	0.17*	0.17*	−0.13	0.27
LPA	−0.25**	0.04	0.09	−0.22**	0.20**	0.17*	−0.15*
MPA	−0.21**	−0.18**	0.01	−0.26**	−0.20**	0.24**	−0.38**
VPA	−0.25**	−0.17*	−0.04	−0.29**	−0.16*	0.19**	−0.39**

* : *p* <0.05, **: *p* <0.01.

CMR-z, cardiometabolic risk Z-score; FINs, fasting insulin; FPG, fasting plsma glucose; HDL-c, high-density lipoprotein cholesterol; LPA, light physical activity; MPA, moderate physical activity; SB, sedentary behavior; TC, total cholestero; TPA: total physical activity; VPA, vigorous physical activity; WC, waist circumferencel.

### 3.5 Effect of 10 min of different intensity physical activity substitution for SB on CMR factors

**FIGURE 2:**
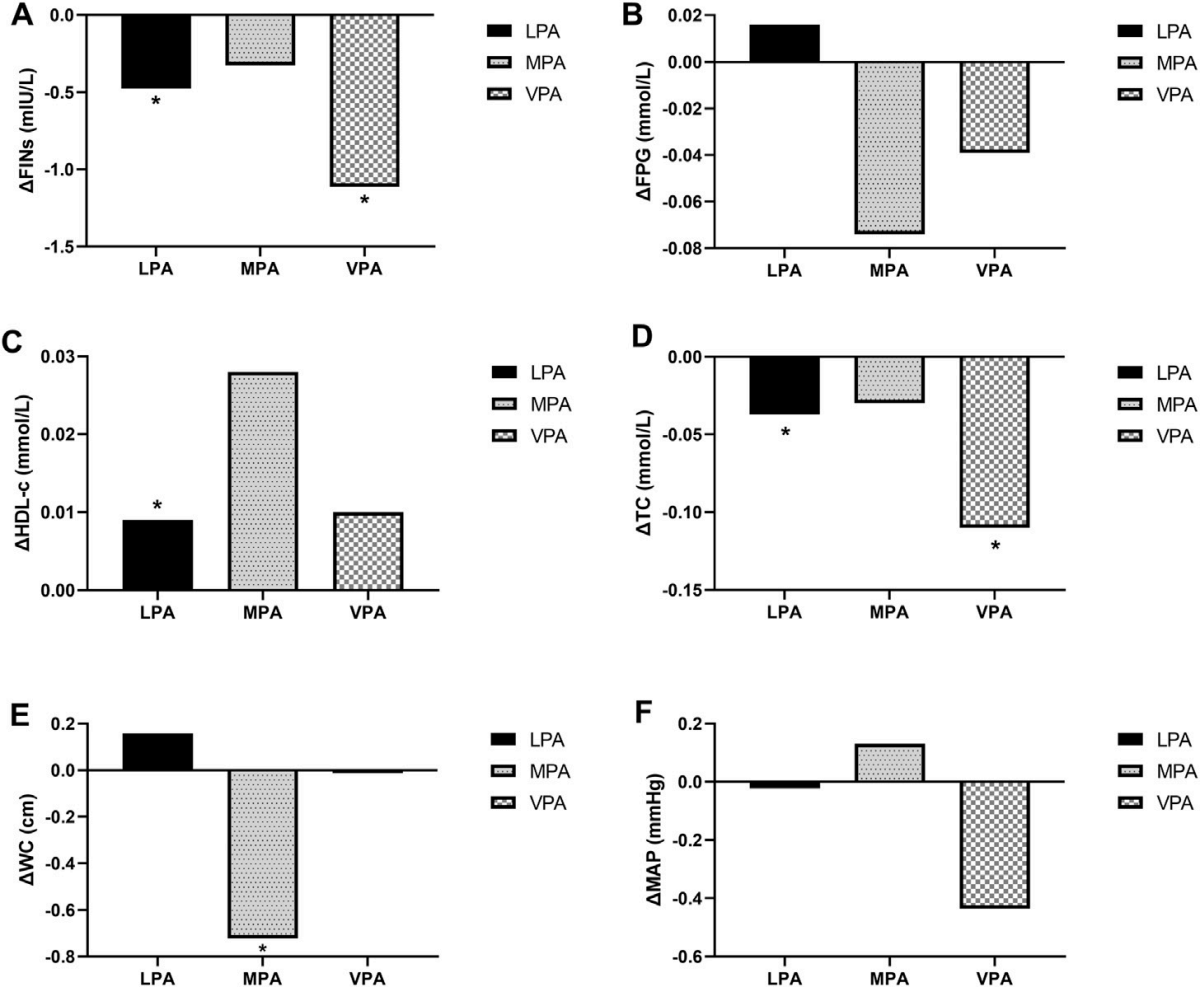
Effect of 10 min of different intensity physical activity substitution for SB on CMR factors: The difference of FINs **(A)**, FPG **(B)**, HDL-c **(C)**, TC **(D)**, WC **(E)**, MAP **(F)**. *, P<0.05. Corrections for gender, age, and REE when building the ISM. FINs, Fasting Insulin; FPG, Fasting Plsma Glucose; HDL-c, High-density Lipoprotein Cholesterol; LPA, Light Physical Activity; MAP, Mean Artery Pressure; MPA, Moderate Physical Activity; TC, Total Cholestero; VPA, Vigorous Physical Activity; WC, Waist Circumferencel.

Based on the ISM at 10 min, LPA, MPA, and VPA were substituted for SB, and the CMR factors were largely effectively improved. LPA improved the blood indexes FINs [β = −0.49 mIU/L, 95% CI (−0.81, −0.17)]; TC [β = −0.04 mmol/L, 95% CI (−0.07, −0.01)]; and HDL-c [β = 0.01 mmol/L, 95% CI (0.01, 0.02)]. VPA improved FINs [β = −1.10 mIU/L, 95% CI (−2.01, −0.188), *p* = 0.02] and TC [β = −0.11 mmol/L, 95% CI (−0.20, −0.02)]; and MPA improved WC [β = −0.69 cm, 95% CI (−1.28, −0.09)]. MAP and FPG were not changed (Figures 2A–F).

### 3.6 Effects of different intensity physical activity substitutions for SB on ΔCMR-z under different models

Under the single model, 10 min of SB led to an increase in CMR-z whereas 10 min of LPA (*p* = 0.02), MPA (*p* <0.01), and VPA (*p* <0.01) reduced CMR-z levels, and MPA had the most significant effect. However, in the partition model, only VPA reduced CMR-z levels [β = −0.34, 95% CI (−0.62, −0.07)]. SB led to an increase in CMR-z levels, and LPA and MPA reduced CMR-z, but none of the effects were significant (*p* >0.05) (see Table 5).

**TABLE 5:** Effects of three different models on ΔCMR-z after substitution for SB with different intensity physical activity.

		SB		LPA		MPA		VPA	
		β(95%CI)	*P*	β(95%CI)	*P*	β(95%CI)	*P*	β(95%CI)	*P*
SM		0.12 (0.05,0.18)	<0.01	−0.09 (−0.17,−0.02)	0.02	−0.64 (−0.87,−0.42)	<0.01	−0.60 (−0.80,−0.39)	<0.01
PM		0.05 (−0.01,0.11)	0.13	−0.05 (−0.13, .02)	0.13	−0.27 (−0.58, 0.04)	0.09	−0.34 (−0.62,-0.07)	0.02
ISM	SB	Dropped		−0.10 (−0.20,−0.01)	0.04	−0.32 (−0.63,−0.01)	0.04	−0.39 (−0.66,−0.20)	0.01
	LPA	0.10 (0.01,0.12)	0.04	Dropped		−0.22 (−0.55, 0.12)	0.20	−0.29 (−0.57,-0.01)	0.05
	MPA	0.32 (0.01,0.63)<	0.04	0.22 (−0.12,0.55)	0.20	Dropped		−0.07 (−0.60,0.46)<	0.79
	VPA	0.39 (0.12,0.66)	0.01	0.29 (0.01, 0.57)	0.05	0.07 (−0.46, 0.60)	0.79	Dropped	

Correction for gender, age, and REE, when building the SM, PM, ISM.

CI, confidence interva; ISM, isotemporal substitution model; LPA, light physical activity; MPA, moderate physical activity; SB, sedentary behavior; SM, single model; PM, partition model; VPA, vigorous physical activity; TPA, total physical activity.

Under the ISM, 10 min of LPA reduced CMR-z [β = −0.10, 95% CI (−0.20, −0.01)]; 10 min of MPA also reduced CMR-z [β = −0.32, 95% CI (−0.63, −0.01)]; 10 min of VPA reduced CMR-z [β = −0.39, 95% CI (−0.66, −0.12)] (see Table 5).

### 3.7 Effect of 20 min, 30 min, 40 min, 50 min, and 60 min of LPA, MPA, or VPA substitution for SB on CMR-z

**FIGURE 3:**
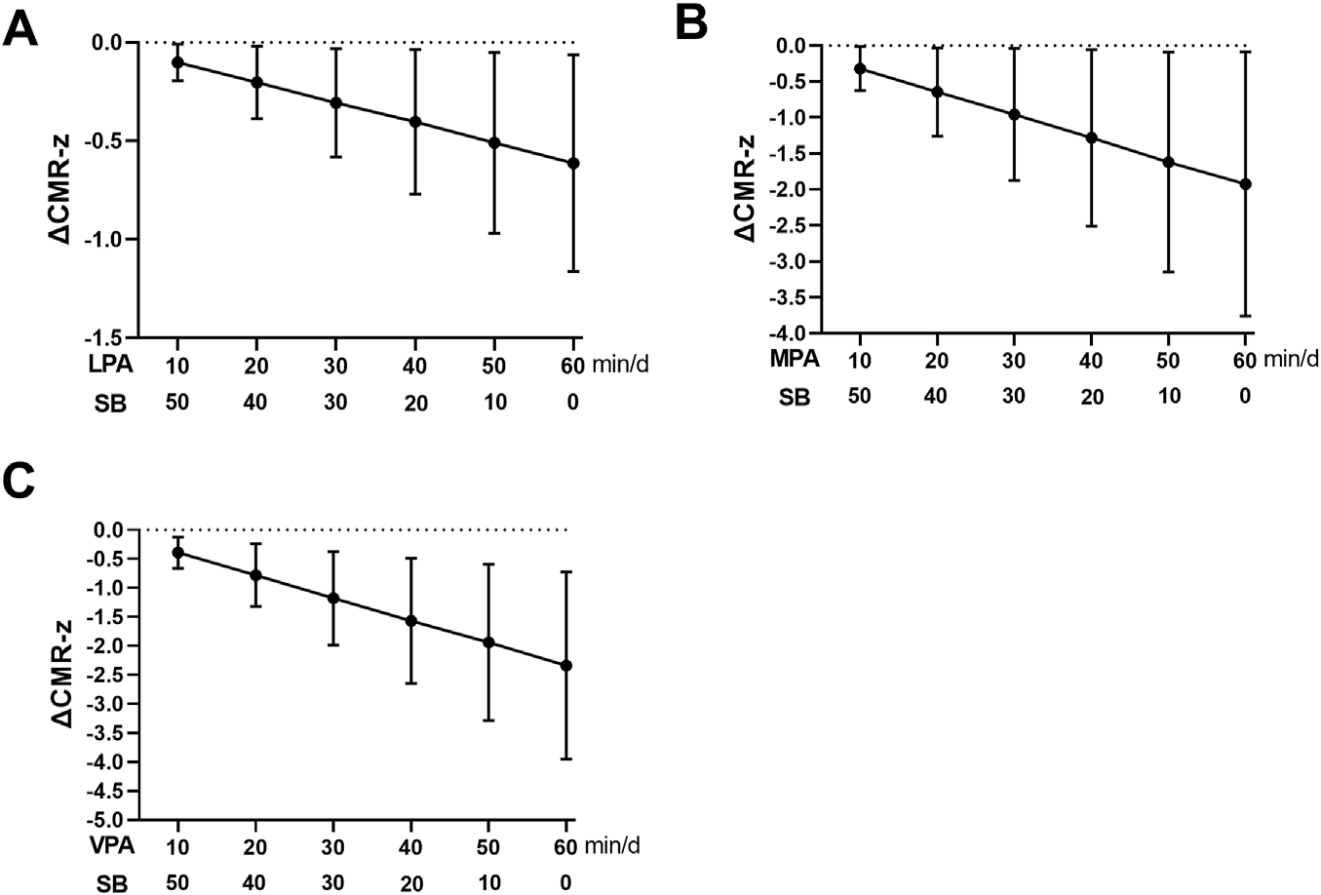
Effect of 20 min, 30 min, 40 min, 50 min and 60 min LPA **(A)**, MPA **(B)**, VPA **(C)** substitution for SB on CMR-z. CMR-z, Cardiometabolic risk Z-score; LPA, light physical activity; MPA, moderate physical activity; SB, sedentary behavior; VPA, vigorous physical activity.

The improvement in CMR-z was further amplified after substituting for SB for 20 min, 30 min, 40 min, 50 min, and 60 min of LPA, MPA, and VPA. The ΔCMR-z was [β = −0.31, 95% CI (−0.58, −0.03)] when using 30 min of LPA for substitution and [β = −0.61, 95% CI (−1.16, −0.06)] at 60 min ΔCMR-z was [β = −0.96, 95% CI (−1.88, −0.04)] when using 30 min of MPA for substitution and [β = −1.92, 95% CI (−3.76, −0.09)] at 60 min ΔCMR-z was [β = −1.18, 95% CI (−1.99, −0.37)] when using 30 min of VPA for substitution and [β = −2.34, 95% CI (−3.95, −0.72)] at 60 min (Figures 3A–C).

## 4 Discussion

The main findings of this study were as follows: 1) 10 min of LPA, MPA, and VPA per day were effective in reducing CMR-z in obese children and adolescents after substituting them for SB, which further verified that reducing SB was effective in reducing cardiovascular risk in obese children; 2) there was no significant reduction in CMR-z after replacing LPA with MPA for 10 min per day whereas 10 min of VPA replacing LPA significantly decreased CMR-z; however, MPA and VPA did not show significant improvement in CMR-z after replacing each other. This suggests that increasing the duration of MPA and VPA and reducing SB are effective in reducing cardiovascular risk and that MPA and VPA are more effective than LPA in improving cardiovascular risk in obese children. For cardiovascular disease factors, the differences were not significant between LPA, MPA, and VPA, and longer intervention duration will be conducted.

The 24 h of a day mainly includes sleep, SB, LPA, MPA, and VPA, thus adding more of one activity requires a corresponding reduction of another one (Chastin et al., 2015). Studies of obese children and adolescents have found that when the daily SB duration is longer, the LPA, and MVPA duration will be shorter (Schwarzfischer et al., 2019). Previous research models on cardiovascular risk in obese children have explored the effects of CMR and cardiovascular risk factors after simply increasing the duration of LPA, MPA, or VPA (i.e., single model), and this statistical analysis method does not consider that increasing the duration of LPA, MPA, or VPA alone may amplify the effects of the intervention (consistent with the single model results of this study). By contrast, partition model analysis found no significant effect of SB, LPA, and MPA on CMR-z whereas only VPA was effective in reducing CMR-z. When partition model analysis was performed, because TPA was not included in the model, its coefficient indicated only the effect of increasing the duration of that type of PA on CMR-z, which can lead to contradictory results due to the interaction between variables.

In this study, the ISM revealed that substituting 10 min of LPA, MPA, and VPA for SB was effective in reducing CMR-z in obese children and adolescents. Further analysis revealed that VPA may have the best intervention effect. In addition, the significant reduction in CMR-z after SB replacement with LPA suggests that for obese children and adolescents, the primary goal of improving cardiometabolic health is to reduce SB and increase physical activity.

SB is mainly defined as a quiet, awake activity in a sitting or lying position; metabolic equivalents ≤1.4 METs (Tremblay et al., 2017). One study found that children and adolescents spend up to 400 min/d in sedentary hypermobility, including 150 min of screen time (Barnett et al., 2018). SB has a strong positive correlation with obesity and increases the risk of developing cardiovascular risk factors such as T2DM, hypertension, and dyslipidemia in childhood (Carson et al., 2016). The possible mechanisms by which SB leads to elevated cardiovascular risk in the organism are mainly hemodynamic stimulation (Johnson et al., 2011), chronic inflammatory response (Howard et al., 2015), and metabolic marker aggregation (Carter et al., 2017). Therefore, reducing SB is essential to reducing cardiovascular risk.

Although studies have confirmed that increasing moderate-to-vigorous physical activity is essential for reducing cardiovascular risk, most current studies are based on adults (Giada et al., 2008; Han et al., 2018). For children and adolescents, the improvement effects of different intensities of physical activity are poorly understood. Although current evidence does not yet precisely confirm the differences between the improvement effects of different intensities, it appears that the longer the duration of MVPA, the more significant the health benefits (Carson et al., 2013). Ekelund et al. (2012)) found from a pooled analysis of 14 independent studies that longer daily MVPA time was associated with lower levels of cardiovascular risk factors such as WC, blood pressure, TC, and FINs in children and adolescents. Knaeps et al. (2018) also showed that reducing sedentary and less-active time and increasing MVPA activity time were effective in improving cardiovascular health and reducing cardiovascular risk. Both the American Academy of Pediatrics and the WHO recommend that children and adolescents participate in at least 60 min of MVPA and less than 120 min of screen time daily (Barlow and Expert Committee, 2007; Bull et al., 2020). The European Society of Endocrinology and the Pediatric Endocrine Society recommend that obese children and adolescents reduced inactivity time and increase a minimum of 20 min of MVPA daily, with a goal of 60 min (Styne et al., 2017). Therefore, increasing MPA and VPA levels is essential to reducing cardiovascular risk in obese children.

Although MVPA is essential for improving cardiometabolic health, the volume of physical activity diminishes with increasing intensity, and LPA is an important component of daily physical activity (Carson et al., 2013). Therefore, the findings from this study have potentially important public health implications. Although participating in MVPA for 60 min or more per day remains the ideal goal, this may not be realistic for some chindren and adolescents. LPA is easier to achieve and promote than MVPA, especially among obese pediatric adolescents who do not meet physical activity recommendations.

Research studies have found that the rate of physical inactivity (less than 60 min of MVPA per day) among students aged 11–17 years is as high as 81.0% (Guthold et al., 2020), which is an important contributing factor to the increased incidence of cardiovascular disease in children. In particular, the impact of measures such as segregation, home study, and elimination of group sports since the COVID-19 pandemic has also led to increased physical inactivity in children and adolescents (Trott et al., 2022). Evidence suggests that more than half of SB time among children and adolescents occurs after school (Arundell et al., 2016), so increasing after-school PA levels is important in improving PA deficits (Daly et al., 2021; Neil-Sztramko et al., 2021). This also suggests the need for future school, community, and clinical strategies to improve cardiovascular health in obese children (Ainsworth, 2016).

## 5 Strengths and limitations

The subjects of this study were obese children and adolescents who attended summer camps, during which there were planned exercise training, standardized daily management, and avoidance of interference factors, which directly reflected the effects of different intensities of physical activity on cardiovascular risk, which also better informed the development of policies related to physical activity in children and adolescents. However, the sleep duration of the subjects was not included in the model for analysis in this study. Some studies have found that sleep duration may also affect cardiovascular risk factors (Saunders et al., 2016).

## 6 Conclusion

Increasing LPA was effective in improving cardiometabolic risk in obese children, and the more intense the exercise, the more significant the improvement. Increasing the duration of LPA, MPA, and VPA can further improve the effect.

## Data Availability

The raw data supporting the conclusion of this article will be made available by the authors, without undue reservation.

## References

[B1] Ainsworth B. E. (2016). How physically active are our children? A global view. J. Sport Health Sci. 5 (4), 400–401. 10.1016/j.jshs.2016.12.003 30356582 PMC6188924

[B2] American Diabetes Association (2017). 12. Children and adolescents. Diabetes Care 40 (1), S105–S113. 10.2337/dc17-S015 27979899

[B3] Andersen L. B. Harro M. Sardinha L. B. Froberg K. Ekelund U. Brage S. (2006). Physical activity and clustered cardiovascular risk in children: A cross-sectional study (the European youth heart study). Lancet 368 (9532), 299–304. 10.1016/S0140-6736(06)69075-2 16860699

[B4] Arundell L. Fletcher E. Salmon J. Veitch J. Hinkley T. (2016). A systematic review of the prevalence of sedentary behavior during the after-school period among children aged 5-18 years. Int. J. Behav. Nutr. Phys. Act. 13 (1), 93. 10.1186/s12966-016-0419-1 27549588 PMC4994288

[B5] Barlow S. E. Expert Committee (2007). Expert committee recommendations regarding the prevention, assessment, and treatment of child and adolescent overweight and obesity: Summary report. Pediatrics 120 (4), S164–S192. 10.1542/peds.2007-2329C 18055651

[B6] Barnett T. A. Kelly A. S. Young D. R. Perry C. K. Pratt C. A. Edwards N. M. (2018). Sedentary behaviors in today's youth: Approaches to the prevention and management of childhood obesity: A scientific statement from the American heart association. Circulation 138 (11), e142–e159. 10.1161/CIR.0000000000000591 30354382

[B7] Berenson G. S. Bogalusa Heart Study group (2012). Health consequences of obesity. Pediatr. Blood Cancer 58 (1), 117–121. 10.1002/pbc.23373 22076834

[B8] Bull F. C. Al-Ansari S. S. Biddle S. Borodulin K. Buman M. P. Cardon G. (2020). World Health Organization 2020 guidelines on physical activity and sedentary behaviour. Br. J. Sports Med. 54 (24), 1451–1462. 10.1136/bjsports-2020-102955 33239350 PMC7719906

[B9] Buman M. P. Winkler E. A. Kurka J. M. Hekler E. B. Baldwin C. M. Owen N. (2014). Reallocating time to sleep, sedentary behaviors, or active behaviors: Associations with cardiovascular disease risk biomarkers, NHANES 2005-2006. Am. J. Epidemiol. 179 (3), 323–334. 10.1093/aje/kwt292 24318278

[B10] Carson V. Hunter S. Kuzik N. Gray C. E. Poitras V. J. Chaput J. P. (2016). Systematic review of sedentary behaviour and health indicators in school-aged children and youth: An update. Appl. Physiol. Nutr. Metab. 41 (3), S240–S265. 10.1139/apnm-2015-0630 27306432

[B11] Carson V. Ridgers N. D. Howard B. J. Winkler E. A. Healy G. N. Owen N. (2013). Light-intensity physical activity and cardiometabolic biomarkers in US adolescents. PLoS One 8 (8), e71417. 10.1371/journal.pone.0071417 23951157 PMC3739773

[B12] Carter S. Hartman Y. Holder S. Thijssen D. H. Hopkins N. D. (2017). Sedentary behavior and cardiovascular disease risk: Mediating mechanisms. Exerc Sport Sci. Rev. 45 (2), 80–86. 10.1249/JES.0000000000000106 28118158

[B13] Chaput J. P. Willumsen J. Bull F. Chou R. Ekelund U. Firth J. (2020). 2020 WHO guidelines on physical activity and sedentary behaviour for children and adolescents aged 5-17 years: Summary of the evidence. Int. J. Behav. Nutr. Phys. Act. 17 (1), 141. 10.1186/s12966-020-01037-z 33239009 PMC7691077

[B14] Chastin S. F. Palarea-Albaladejo J. Dontje M. L. Skelton D. A. (2015). Combined effects of time spent in physical activity, sedentary behaviors and sleep on obesity and cardio-metabolic health markers: A novel compositional data analysis approach. PLoS One 10 (10), e0139984. 10.1371/journal.pone.0139984 26461112 PMC4604082

[B15] Cliff D. P. Jones R. A. Burrows T. L. Morgan P. J. Collins C. E. Baur L. A. (2014). Volumes and bouts of sedentary behavior and physical activity: Associations with cardiometabolic health in obese children. Obes. (Silver Spring) 22 (5), E112–E118. 10.1002/oby.20698 24788574

[B16] Collaborators G. B. D. O. Afshin A. Forouzanfar M. H. Reitsma M. B. Sur P. Estep K. (2017). Health effects of overweight and obesity in 195 countries over 25 years. N. Engl. J. Med. 377 (1), 13–27. 10.1056/NEJMoa1614362 28604169 PMC5477817

[B17] Cooper A. R. Goodman A. Page A. S. Sherar L. B. Esliger D. W. van Sluijs E. M. (2015). Objectively measured physical activity and sedentary time in youth: The international children's accelerometry database (ICAD). Int. J. Behav. Nutr. Phys. Act. 12, 113. 10.1186/s12966-015-0274-5 26377803 PMC4574095

[B18] Daly N. J. Parsons M. Blondino C. Clifford J. S. Prom-Wormley E. (2021). Association between caregiver depression and child after-school program participation. J. Fam. Soc. Work 24 (3), 245–260. 10.1080/10522158.2020.1824954 34239279 PMC8259540

[B19] Drozdz D. Alvarez-Pitti J. Wójcik M. Borghi C. Gabbianelli R. Mazur A. (2021). Obesity and cardiometabolic risk factors: From childhood to adulthood. Nutrients 13 (11), 4176. 10.3390/nu13114176 34836431 PMC8624977

[B20] Ekelund U. Anderssen S. A. Froberg K. Sardinha L. B. Andersen L. B. Brage S. (2007). Independent associations of physical activity and cardiorespiratory fitness with metabolic risk factors in children: The European youth heart study. Diabetologia 50 (9), 1832–1840. 10.1007/s00125-007-0762-5 17641870

[B21] Ekelund U. Luan J. Sherar L. B. Esliger D. W. Griew P. Cooper A. (2012). Moderate to vigorous physical activity and sedentary time and cardiometabolic risk factors in children and adolescents. Jama 307 (7), 704–712. 10.1001/jama.2012.156 22337681 PMC3793121

[B22] Ekelund U. Steene-Johannessen J. Brown W. J. Fagerland M. W. Owen N. Powell K. E. (2016). Does physical activity attenuate, or even eliminate, the detrimental association of sitting time with mortality? A harmonised meta-analysis of data from more than 1 million men and women. Lancet 388 (10051), 1302–1310. 10.1016/S0140-6736(16)30370-1 27475271

[B23] Giada F. Biffi A. Agostoni P. Anedda A. Belardinelli R. Carlon R. (2008). Exercise prescription for the prevention and treatment of cardiovascular diseases: Part I. J. Cardiovasc Med. Hagerst. 9 (5), 529–544. 10.2459/JCM.0b013e3282f7ca77 18404008

[B24] GoCOT, Force (2004). Body mass index reference norm for screening overweight and obesity in Chinese children and adolescents. Zhonghua Liu Xing Bing Xue Za Zhi 25 (2), 97–102. 15132858

[B25] Gortmaker S. L. Must A. Perrin J. M. Sobol A. M. Dietz W. H. (1993). Social and economic consequences of overweight in adolescence and young adulthood. N. Engl. J. Med. 329 (14), 1008–1012. 10.1056/NEJM199309303291406 8366901

[B26] Guthold R. Stevens G. A. Riley L. M. Bull F. C. (2020). Global trends in insufficient physical activity among adolescents: A pooled analysis of 298 population-based surveys with 1·6 million participants. Lancet Child. Adolesc. Health 4 (1), 23–35. 10.1016/S2352-4642(19)30323-2 31761562 PMC6919336

[B27] Han C. Liu F. Yang X. Chen J. Li J. Cao J. (2018). Ideal cardiovascular health and incidence of atherosclerotic cardiovascular disease among Chinese adults: The China-par project. Sci. China Life Sci. 61 (5), 504–514. 10.1007/s11427-018-9281-6 29721777

[B28] Howard B. J. Balkau B. Thorp A. A. Magliano D. J. Shaw J. E. Owen N. (2015). Associations of overall sitting time and TV viewing time with fibrinogen and C reactive protein: The AusDiab study. Br. J. Sports Med. 49 (4), 255–258. 10.1136/bjsports-2013-093014 24550208

[B29] Järvisalo M. J. Rönnemaa T. Volanen I. Kaitosaari T. Kallio K. Hartiala J. J. (2002). Brachial artery dilatation responses in healthy children and adolescents. Am. J. Physiol. Heart Circ. Physiol. 282 (1), H87–H92. 10.1152/ajpheart.2002.282.1.H87 11748051

[B30] Johnson B. D. Mather K. J. Wallace J. P. (2011). Mechanotransduction of shear in the endothelium: Basic studies and clinical implications. Vasc. Med. 16 (5), 365–377. 10.1177/1358863X11422109 22003002

[B31] King W. C. Chen J. Y. Courcoulas A. P. Mitchell J. E. Wolfe B. M. Patterson E. J. (2016). Objectively-measured sedentary time and cardiometabolic health in adults with severe obesity. Prev. Med. 84, 12–18. 10.1016/j.ypmed.2015.12.007 26724517 PMC4758881

[B32] Kit B. K. Kuklina E. Carroll M. D. Ostchega Y. Freedman D. S. Ogden C. L. (2015). Prevalence of and trends in dyslipidemia and blood pressure among US children and adolescents, 1999-2012. JAMA Pediatr. 169 (3), 272–279. 10.1001/jamapediatrics.2014.3216 25599372 PMC7423159

[B33] Knaeps S. Bourgois J. G. Charlier R. Mertens E. Lefevre J. Wijndaele K. (2018). Ten-year change in sedentary behaviour, moderate-to-vigorous physical activity, cardiorespiratory fitness and cardiometabolic risk: Independent associations and mediation analysis. Br. J. Sports Med. 52 (16), 1063–1068. 10.1136/bjsports-2016-096083 27491779 PMC6089204

[B34] Larouche R. Garriguet D. Tremblay M. S. (2017). Outdoor time, physical activity and sedentary time among young children: The 2012-2013 Canadian Health Measures Survey. Can. J. Public Health 107 (6), e500–e506. 10.17269/cjph.107.5700 28252366 PMC6972245

[B35] Larouche R. Mire E. F. Belanger K. Barreira T. V. Chaput J. P. Fogelholm M. (2019). Relationships between outdoor time, physical activity, sedentary time, and body mass index in children: A 12-country study. Pediatr. Exerc Sci. 31 (1), 118–129. 10.1123/pes.2018-0055 30304983

[B36] Lavie C. J. Ozemek C. Carbone S. Katzmarzyk P. T. Blair S. N. (2019). Sedentary behavior, exercise, and cardiovascular health. Circ. Res. 124 (5), 799–815. 10.1161/CIRCRESAHA.118.312669 30817262

[B37] Lemos L. Clark C. Brand C. Pessoa M. L. Gaya A. Mota J. (2021). 24-hour movement behaviors and fitness in preschoolers: A compositional and isotemporal reallocation analysis. Scand. J. Med. Sci. Sports 31 (6), 1371–1379. 10.1111/sms.13938 33599022

[B38] Lightwood J. Bibbins-Domingo K. Coxson P. Wang Y. C. Williams L. Goldman L. (2009). Forecasting the future economic burden of current adolescent overweight: An estimate of the coronary heart disease policy model. Am. J. Public Health 99 (12), 2230–2237. 10.2105/AJPH.2008.152595 19833999 PMC2775763

[B39] Mekary R. A. Willett W. C. Hu F. B. Ding E. L. (2009). Isotemporal substitution paradigm for physical activity epidemiology and weight change. Am. J. Epidemiol. 170 (4), 519–527. 10.1093/aje/kwp163 19584129 PMC2733862

[B40] Molnár D. Jeges S. Erhardt E. Schutz Y. (1995). Measured and predicted resting metabolic rate in obese and nonobese adolescents. J. Pediatr. 127 (4), 571–577. 10.1016/s0022-3476(95)70114-1 7562278

[B41] Neil-Sztramko S. E. Caldwell H. Dobbins M. LaRocca R. L. (2021). School-based physical activity programs for promoting physical activity and fitness in children and adolescents aged 6 to 18. Cochrane Database Syst. Rev. 9 (9), Cd007651. 10.1002/14651858.CD007651.pub2 34555181 PMC8459921

[B42] Physical status (1995). Physical status: The use and interpretation of anthropometry. Report of a WHO Expert committee. World Health Organ Tech. Rep. Ser. 854, 1–452. 8594834

[B43] Sardinha L. B. Marques A. Minderico C. Ekelund U. (2017). Cross-sectional and prospective impact of reallocating sedentary time to physical activity on children's body composition. Pediatr. Obes. 12 (5), 373–379. 10.1111/ijpo.12153 27256488 PMC6258907

[B44] Saunders T. J. Gray C. E. Poitras V. J. Chaput J. P. Janssen I. Katzmarzyk P. T. (2016). Combinations of physical activity, sedentary behaviour and sleep: Relationships with health indicators in school-aged children and youth. Appl. Physiol. Nutr. Metab. 41 (3), S283–S293. 10.1139/apnm-2015-0626 27306434

[B45] Schwarzfischer P. Gruszfeld D. Stolarczyk A. Ferre N. Escribano J. Rousseaux D. (2019). Physical activity and sedentary behavior from 6 to 11 years. Pediatrics 143 (1), e20180994. 10.1542/peds.2018-0994 30509928

[B46] Skrede T. Stavnsbo M. Aadland E. Aadland K. N. Anderssen S. A. Resaland G. K. (2017). Moderate-to-vigorous physical activity, but not sedentary time, predicts changes in cardiometabolic risk factors in 10-y-old children: The active smarter kids study. Am. J. Clin. Nutr. 105 (6), 1391–1398. 10.3945/ajcn.116.150540 28381476

[B47] Skrede T. Steene-Johannessen J. Anderssen S. A. Resaland G. K. Ekelund U. (2019). The prospective association between objectively measured sedentary time, moderate-to-vigorous physical activity and cardiometabolic risk factors in youth: A systematic review and meta-analysis. Obes. Rev. 20 (1), 55–74. 10.1111/obr.12758 30270500

[B48] Styne D. M. Arslanian S. A. Connor E. L. Farooqi I. S. Murad M. H. Silverstein J. H. (2017). Pediatric obesity-assessment, treatment, and prevention: An endocrine society clinical practice guideline. J. Clin. Endocrinol. Metab. 102 (3), 709–757. 10.1210/jc.2016-2573 28359099 PMC6283429

[B49] Sun Y. Yin X. Li Y. Bi C. Li M. Yang X. (2020). Isotemporal substitution of sedentary behavior for physical activity on cardiorespiratory fitness in children and adolescents. Med. Baltim. 99 (30), e21367. 10.1097/MD.0000000000021367 PMC7386960 32791744

[B50] Tabachnick B. G. Fidell L. S. (2007). Using multivariate statistics. 5th ed.

[B51] Tirosh A. Shai I. Afek A. Dubnov-Raz G. Ayalon N. Gordon B. (2011). Adolescent BMI trajectory and risk of diabetes versus coronary disease. N. Engl. J. Med. 364 (14), 1315–1325. 10.1056/NEJMoa1006992 21470009 PMC4939259

[B52] Tounian P. Aggoun Y. Dubern B. Varille V. Guy-Grand B. Sidi D. (2001). Presence of increased stiffness of the common carotid artery and endothelial dysfunction in severely obese children: A prospective study. Lancet 358 (9291), 1400–1404. 10.1016/S0140-6736(01)06525-4 11705484

[B53] Tremblay M. S. Aubert S. Barnes J. D. Saunders T. J. Carson V. Latimer-Cheung A. E. (2017). Sedentary behavior research network (SBRN) - terminology consensus project process and outcome. Int. J. Behav. Nutr. Phys. Act. 14 (1), 75. 10.1186/s12966-017-0525-8 28599680 PMC5466781

[B54] Trott M. Driscoll R. Irlado E. Pardhan S. (2022). Changes and correlates of screen time in adults and children during the COVID-19 pandemic: A systematic review and meta-analysis. EClinicalMedicine 48, 101452. 10.1016/j.eclinm.2022.101452 35615691 PMC9122783

[B55] Väistö J. Eloranta A. M. Viitasalo A. Tompuri T. Lintu N. Karjalainen P. (2014). Physical activity and sedentary behaviour in relation to cardiometabolic risk in children: Cross-sectional findings from the physical activity and nutrition in children (PANIC) study. Int. J. Behav. Nutr. Phys. Act. 11, 55. 10.1186/1479-5868-11-55 24766669 PMC4008488

[B56] Väistö J. Haapala E. A. Viitasalo A. Schnurr T. M. Kilpeläinen T. O. Karjalainen P. (2019). Longitudinal associations of physical activity and sedentary time with cardiometabolic risk factors in children. Scand. J. Med. Sci. Sports 29 (1), 113–123. 10.1111/sms.13315 30276872 PMC6485341

[B57] Wang J. Thornton J. C. Kolesnik S. Pierson R. N. Jr (2000). Anthropometry in body composition. An overview. Ann. N. Y. Acad. Sci. 904, 317–326. 10.1111/j.1749-6632.2000.tb06474.x 10865763

[B58] WHO Guidelines Approved by the Guidelines Review Committee (2010). WHO Guidelines approved by the Guidelines Review committee. Geneva: World Health Organization Copyright © World Health Organization 2010.

[B59] Zhu L. Chen P. J. (2012). Verification of the self-reported pubertal development scale(Chinese version). Chin. J. Sports Med. 31 (06), 512–516.

[B60] Zhu Z. Tang Y. Zhuang J. Liu Y. Wu X. Cai Y. (2019). Physical activity, screen viewing time, and overweight/obesity among Chinese children and adolescents: An update from the 2017 physical activity and fitness in China-the youth study. BMC Public Health 19 (1), 197. 10.1186/s12889-019-6515-9 30767780 PMC6376726

